# Anaplastic lymphoma kinase gene rearrangements in patients with advanced-stage non-small-cell lung cancer: CT characteristics and response to chemotherapy

**DOI:** 10.1002/cam4.172

**Published:** 2013-12-27

**Authors:** Jangchul Park, Hidekazu Yamaura, Yasushi Yatabe, Waki Hosoda, Chiaki Kondo, Junichi Shimizu, Yoshitsugu Horio, Kimihide Yoshida, Kosuke Tanaka, Tomoyo Oguri, Yoshihisa Kobayashi, Toyoaki Hida

**Affiliations:** 1 Department of Thoracic Oncology, Aichi Cancer Center HospitalNagoya, 464-8681, Japan; 2 Department of Diagnostic and Interventional Radiology, Aichi Cancer Center HospitalNagoya, 464-8681, Japan; 3 Department of Pathology and Molecular Diagnostics, Aichi Cancer Center HospitalNagoya, 464-8681, Japan; 4 Department of Thoracic Surgery, Aichi Cancer Center HospitalNagoya, 464-8681, Japan

**Keywords:** Anaplastic lymphoma kinase, chemotherapy, computed tomography, ground-glass opacity, lung cancer

## Abstract

Few articles have been published on the imaging findings of anaplastic lymphoma kinase (*ALK*)-positive non-small-cell lung cancer (NSCLC). To investigate the radiological findings of *ALK*-positive NSCLC in the advanced stage, CT scans were examined. In addition, the response to chemotherapy was evaluated. Of the 36 patients with *ALK*-rearranged NSCLC, a mass and a nodule were identified in 17 (47.2%) and 16 (44.4%), respectively, indicating that more than 40% had a small-sized tumor. Overall, 31 (86.1%) patients had lymphadenopathy, seven (19.4%) had extranodal lymph node invasion, and three (8.3%) had lymphangitis. A pleural effusion was seen in 15 patients (41.7%). All but one patient had no ground-glass opacity (GGO) lesions, indicating that most *ALK*-positive tumors showed a solid growth pattern without GGO on CT. Twenty were evaluable for response to chemotherapy; 10 (50.0%) had a partial response (PR), nine (45.0%) had stable disease (SD), and one (5.0%) had progressive disease (PD) with first-line chemotherapy. With second-line chemotherapy, five (26.3%) had PR, 11 (57.9%) had SD, and three (15.8%) had PD. The five patients with PR were all treated by using crizotinib. Time to progression was 8.2 months with first-line chemotherapy, and 6.0 months with second-line chemotherapy. Advanced-stage *ALK*-positive tumors have a relatively aggressive phenotype, which cannot be inferred from the size of the tumor alone. *ALK*-positive patients have a good response to first-line cytotoxic drugs and to crizotinib as second-line therapy, but a relatively poor response to cytotoxic drugs as second-line therapy.

## Introduction

*EML4-ALK* is a fusion-type protein tyrosine kinase that is present in ∼5% of cases of non-small-cell lung cancer (NSCLC). It is generated as a result of a small inversion within the short arm of human chromosome 2. *EML4-ALK* fusion genes have been observed predominantly in adenocarcinomas, younger patients, and never/light smoker patients 1,2. In the phase I trial of crizotinib, a remarkable response rate was observed specifically in anaplastic lymphoma kinase (*ALK*)-positive NSCLC patients 3. As *EML4-ALK* fusion is not as frequent as *EGFR* gene mutation, it would be important to efficiently and accurately identify those lung adenocarcinomas that harbor *ALK* rearrangements in clinical practice to guide the appropriate therapy. Although there has been one clinical report about the radiological features of *ALK*-positive patients so far, it was a report of surgically resectable patients at an early stage 4, but advanced unresectable NSCLC patients were not included. In particular, detailed radiological findings in *ALK*-positive NSCLC in the advanced stage have never been reported that would provide important information for clinicians. In this study, the clinicoradiological characteristics of 36 cases of *ALK*-positive NSCLC in the advanced stage are reported.

## Materials and Methods

### Cases

Thirty-six cases of advanced-stage *ALK*-rearranged lung cancer were evaluated. The patients were all treated without surgery at Aichi Cancer Center Hospital between July 2006 and October 2012. The cases were reviewed and staged according to the seventh edition of the American Joint Committee on Cancer manual. Of the 36 cases, the treatment response to anticancer agents was evaluated in 20 cases. Pathological specimens were obtained by transbronchial lung biopsy, endoscopic ultrasound-guided fine-needle biopsy, percutaneous core needle biopsy, and thoracentesis for pleural effusion. Approval for this study was obtained from the Ethics Committee of Aichi Cancer Center (approval number 4–155). This study was conducted according to the amended Declaration of Helsinki, and written informed consent was obtained from all subjects.

### Detection of the *EML4-ALK* gene

**Figure 1:**
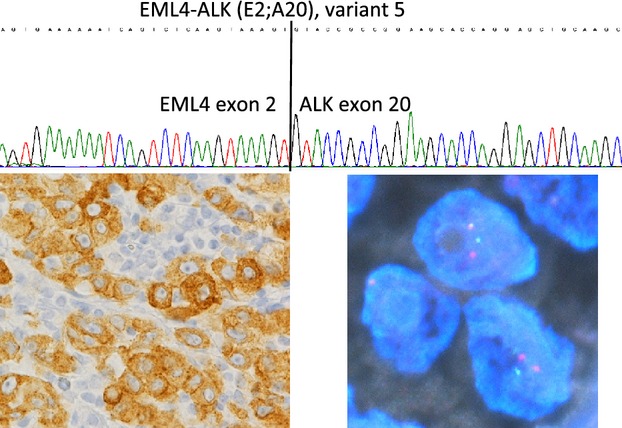
ALK, IHC, FISH, and sequence electropherogram of a lung adenocarcinoma with ALK rearrangement. ALK, anaplastic lymphoma kinase.

Immunohistochemistry or RT-PCR (polymerase chain reaction) was used to screen for *EML4-ALK* fusion. Immunohistochemical analysis was done with the detection system, EnVision FLEX+ (Dako, Glostrup, Denmark), using an autostainer (Dako). The linker in the EnVision Flex+ detection system yields high sensitivity 5. Briefly, 4-*μ*m-thick slides were deparaffinized and pretreated with antigen retrieval solution at a high pH (pH 9.0) using heating instruments (PTlink; Dako). Mouse monoclonal anti-human ALK (clone 5A4, Santa Cruz Biotechnology, Santa Cruz, CA) was reacted for 30 min, and subsequent procedures were followed according to the manufacturer's instructions (Fig. 1). For RT-PCR, multiplex PCR was used according to the procedures reported previously 6 with minor modification. When positive results were obtained with either method, gene rearrangement of *ALK* was confirmed with fluorescent in situ hybridization (Break-Apart Rearrangement Probe; Abbott Molecular Inc., Des Plaines, IL). ALK FISH was considered positive when more than 15% of 100 or more analyzed cells showed splitting of the fluorescent probes according to the manufacturer's criteria (Fig. 1).

### Data collection

All patients' medical records were reviewed to extract data on clinicoradiological characteristics. Tumor response was examined by CT and evaluated according to the Response Evaluation Criteria in Solid Tumors version 1.1. Time to progression (TTP) was measured from the first day of treatment until radiological progression.

### CT examination

CT was performed with 10-, 7-, or 5-mm collimation. All CT scans were reviewed for the presence of a mass (>30 mm in diameter), nodule (≤30 mm in diameter), consolidation, ground-glass opacity (GGO), air bronchograms, adenopathy (defined as hilar, mediastinal, subclavicular, or axillary nodes 10 mm or greater in short-axis dimension), bronchial abnormalities (including wall thickening and bronchial dilatation), pleural effusion, extranodal invasion of lymph nodes, and lymphangitis. Consolidation on CT was defined as increased density of the lung parenchyma with obscuration of the pulmonary vessels. GGO was defined as a hazy increase in attenuation that did not obscure normal lung markings. Air bronchograms on CT were defined as air-filled bronchi seen as radiolucent, branching bands within pulmonary densities. Extranodal invasion was defined as invasion to adjacent tissue. The largest diameter measurements were obtained manually using the picture archiving and communication system measurement electronic tool in all cases. One radiologist (H. Y.) and two chest physicians (J. P. and T. H.) interpreted the chest CT scans and reached their conclusions by consensus.

### Histological analysis

For each case, multiple slides corresponding to tissue sections were reviewed simultaneously by at least two pathologists and classified according to WHO pathological criteria of 2004.

## Results

### Patients' characteristics

Twenty-two patients (61.1%) were women and 14 (38.9%) were men, and their mean age was 48.4 years (range, 26–79 years). Twenty-eight patients were never or light smokers (never smokers smoked <100 cigarettes in their lifetime; light smokers smoked ≤10 pack-years; smokers smoked >10 pack-years), and mean smoking history was 6.5 pack-years (range, 2–59 pack-years). All patients had received a diagnosis of advanced-stage lung adenocarcinoma (4 [11.1%] stage III disease, 32 [88.9%] stage IV disease). The site of metastasis at first onset involved bones in 16 cases (44.4%), brain in nine (25%), opposite lung in seven (19.4%), liver in five (13.9%), extrathoracic lymph nodes in four (11.1%), adrenal gland in two (5.6%), and stomach, choroid, kidney, and spleen in one (2.8%) each.

### Radiological findings

**Figure 2:**
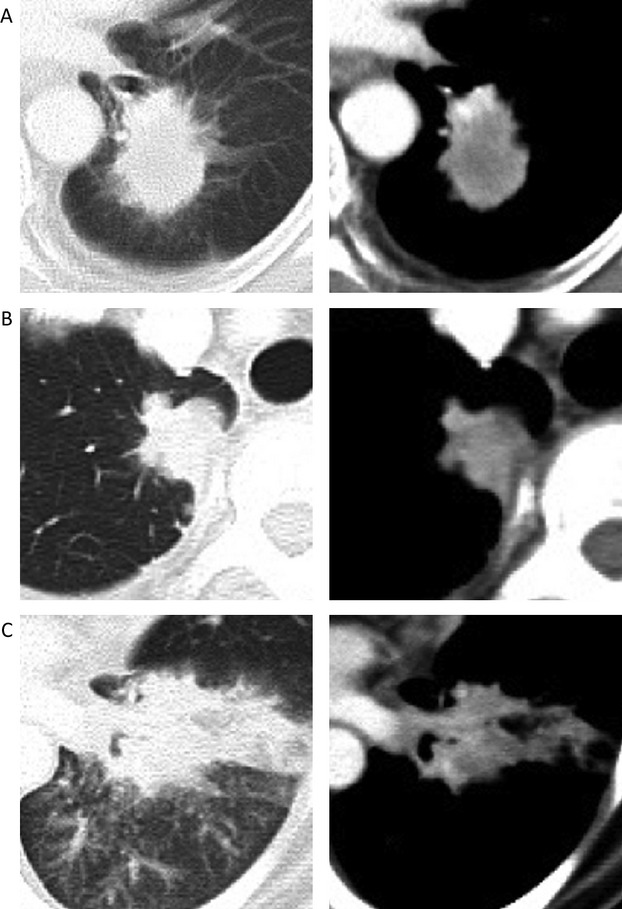
Thoracic CT findings from representative patients in *ALK*-positive NSCLC. (A) mass, (B) nodule, and (C) consolidation. ALK, anaplastic lymphoma kinase; NSCLC, non-small-cell lung cancer.

**Figure 3:**
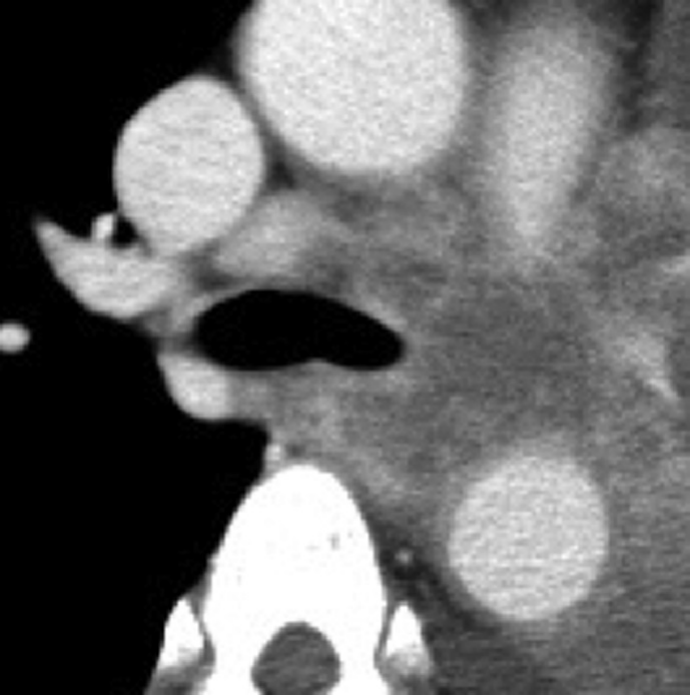
Representative cut of thoracic CT scan showing extranodal invasion of lymph nodes.

Of the 36 patients with *ALK*-positive lung cancer, a mass (Fig. 2A) and a nodule (Fig. 2B) were identified in 17 (47.2%) and 16 (44.4%), respectively, indicating that more than 40% of advanced-stage *ALK*-positive tumors had a small-sized tumor. Consolidations with a peribronchovascular distribution were identified in four of the 36 patients (11.1%, Fig. 2C), and one patient had a nodule and consolidation. In addition, 31 (86.1%) patients had lymphadenopathy, seven (19.4%) patients had extranodal lymph node invasion (Fig. 3), and three (8.3%) patients had lymphangitis. A pleural effusion was seen in 15 patients (41.7%, Table 1). All but one patient had no GGO lesions, indicating that the majority of *ALK*-positive tumors showed a solid growth pattern without GGO on CT.

**Table 1:** Clinical features and CT scan findings of advanced *ALK*-positive lung cancer.

					Chest CT findings					
Case	Gender/age (y)	Smoking history^1^	Stage (TNM)	Detection method for ALK rearrangement	Mass/nodule	Consolidation	Any GGO	Adenopathy hilar/mediastinal/subclavicular	Pleural effusion	Lymphangitis
1	F/57	0	T2bN3M1a—IV	F, I	+/−	−	−	−/+/−	+	−
2	M/57	0	T1bN0M1a—IV	F, I	−/+	−	−	−/−/−	+	−
3	M/66	33	T3N3M1a—IV	F, I	+/−	−	−	+/+/−	+	−
4	F/46	0	T3N2M0—IIIA	F, I	+/−	−	−	−/+/−	−	−
5	F/26	13	T4N3M1b—IV	F, I, P	−/+	−	−	−/−/+	+	−
6	M/32	6	T3N0M1a—IV	F	−/+	+	+	−/−/−	+	+
7	F/57	0	T3N3M1b—IV	F, I	−/+	−	−	−/−/+	−	−
8	F/31	0	T1aN3M1b—IV	F, I	−/+	−	−	+/+/−	−	−
9	F/28	0	T3N3M1b—IV	F	−/−	+	−	+/+/−	−	+
10	F/79	0	T2bN3M0—IIIB	F, I, P	+/−	−	−	+/+/−	−	−
11	M/40	11	T4N3M1b—IV	F	+/−	−	−	−/+/+	−	−
12	F/60	0	T1bN1M1b—IV	F	−/+	−	−	+/−/−	−	−
13	M/59	59	T3N3M1b—IV	F, I	−/−	+	−	+/+/−	−	−
14	F/36	8	T2bN3M1b—IV	F, I	+/−	−	−	+/+/−	−	−
15	M/34	14	T2bN3M1b—IV	F	+/−	−	−	−/+/−	−	−
16	F/37	8	T1aN3M1b—IV	F, I	−/+	−	−	+/+/+	−	−
17	M/38	8	T4N3M1a—IV	F, I, P	+/−	−	−	+/+/−	+	−
18	F/58	0	T1bN2M1a—IV	F, I, P	−/+	−	−	−/+/−	+	−
19	M/41	0	T4N2M1b—IV	F, I	+/−	−	−	+/+/−	+	−
20	M/34	0	T4N1M1b—IV	F, P	+/−	−	−	+/−/−	−	−
21	F/59	0	T4N2M1b—IV	F, I	−/+	−	−	+/+/−	−	−
22	F/54	0	T1bN0M1a—IV	P	−/+	−	−	−/−/−	+	−
23	M/32	7	T4N1M1b—IV	F, I, P	−/−	+	−	+/−/−	−	−
24	F/64	0	T4N3M1b—IV	I, P	+/−	−	−	+/+/−	+	−
25	M/39	13	T3N1M1b—IV	F, I, P	+/−	−	−	+/−/−	+	−
26	F/36	15	T4N1M1b—IV	F, I	+/−	−	−	+/−/−	+	+
27	F/63	5	T2aN2M1b—IV	I, P	+/−	−	−	+/+/−	−	−
28	M/40	22	T1bN3M1b—IV	F, I	−/+	−	−	+/+/−	−	−
29	F/49	0	T4N2M1b—IV	F, I	−/+	−	−	+/+/−	+	−
30	M/56	2	T4N3M1b—IV	F	−/+	−	−	−/+/−	−	−
31	F/57	6	T4N0M1a—IV	F, I	+/−	−	−	−/−/−	−	−
32	M/61	4	T1bN2M0—IIIA	F, I	−/+	−	−	+/+/−	−	−
33	F/62	0	T4N2M1b—IV	F, I, P	+/−	−	−	+/+/−	−	−
34	F/35	0	T2aN3M0—IIIB	F, I, P	+/−	−	−	+/+/+	−	−
35	F/70	0	T1aN0M1a—IV	F, I	−/+	−	−	−/−/−	+	−
36	F/48	0	T1bN2M1b—IV	F, I	−/+	−	−	−/+/−	+	−

GGO, ground-glass opacity; TNM, tumor–node–metastasis; F, FISH; I, IHC; P, PCR; +, present; −, absent.

1 Smoking history: pack-years.

### Response to chemotherapy of advanced ALK-positive lung cancer

The treatment response to anticancer agents or crizotinib was examined in the 20 cases that were evaluable. For first-line chemotherapy, most of the treatment regimens included carboplatin or cisplatin in combination with one or more therapeutic agents, such as pemetrexed, taxanes, or bevacizumab, except for three cases that were treated with oral S-1 or crizotinib. Ten (50.0%) patients had a partial response (PR), nine (45.0%) had stable disease (SD), and one (5.0%) had progressive disease (PD). Of the 10 who had PR, five cases had received taxane-based therapy, four received pemetrexed-based chemotherapy, and one received crizotinib. The second-line treatment regimens included single therapeutic agents such as crizotinib, docetaxel, or pemetrexed, and platinum doublet. Of the 19 evaluable cases in the second-line setting, five (26.3%) had PR, 11 (57.9%) had SD, and three (15.8%) had PD. The five patients who had PR were all treated by crizotinib. None of the 10 patients treated with cytotoxic agents had a marked clinical response. The mean TTP was 8.2 months (range, 0.8–23.1) with first-line therapy and 6.0 months (range, 1.3–25.3) with second-line therapy (Table 2). In the second-line setting, the mean TTP of the nine patients treated with crizotinib was 8.4 months, while the TTP of nine evaluable patients (except one patient who continued chemotherapy) treated with cytotoxic chemotherapy was 3.6 months.

**Table 2:** Treatment responses to anticancer agents or crizotinib.

	First-line			Second-line		
Case	Regimen	Response	TTP (months)	Regimen	Response	TTP (months)
1	CBDCA + PTX	PR	4.4	DTX	PD	1.3
2	CBDCA + PTX	PR	4.7	CBDCA + VNR	SD	3.1
3	CBDCA + S-1	SD	5.8	DTX	PD	1.4
4	CBDCA + PTX + RT	PR	18.1	DTX	SD	4.9
5	CBDCA + PTX	PR	6.8	Crizotinib	PR	25.3
6	CDDP + DTX	PR	7.6	PEM	SD	4.0
7	CBDCA + PEM	SD	7.2	DTX	SD	5.4
8	CDDP/CBDCA^1^ + PEM	SD	3.4	Crizotinib	PD	1.5
9	CDDP + PEM	PR	7.5	DTX	SD	2.6
10	S-1	SD	23.1	PEM	SD	7.7
11	CBDCA + PTX	SD	6.3	PEM	SD	1.6
12	CDDP + PEM^2^	PR	6.9	Crizotinib	SD	5.5
13	CBDCA + DTX + BEV	SD	2.6	Crizotinib	PR	5.6
14	CDDP + PEM	PR	8.3	Crizotinib	PR	7.8
15	CBDCA + PEM	SD	6.3	Crizotinib	SD	4.9
16	CBDCA + PEM	PR	13.2	Crizotinib	PR	4.6
17	CBDCA + PEM	PD	0.8	Crizotinib	PR	10.3
18	Crizotinib	PR	10.9	BSC	NA	NA
19	Crizotinib	SD	15.8	CDDP + PEM^2^	SD	>3.8
20	CDDP + PEM	SD	4.0	Crizotinib	SD	10.0

CBDCA, carboplatin; CDDP, cisplatin; PTX, paclitaxel; DTX, docetaxel; PEM, pemetrexed; VNR, vinorelbine; BEV, bevacizumab; S-1, oral fluorouracil anticancer drug; RT, radiation therapy; PR, partial response; SD, stable disease; PD, progressive disease; TTP, Time to progression; BSC, best supportive care; NA, not applicable.

1 Cisplatin was used only for one cycle, followed by carboplatin.

2 Followed by maintenance pemetrexed therapy.

## Discussion

*EML4-ALK* fusion was recently identified as a novel molecular abnormality in about 5% of lung adenocarcinomas 7,8. Because *EML4-ALK* fusion is not as frequent as *EGFR* gene mutation, it would be important to efficiently and accurately identify those lung adenocarcinomas that harbor *ALK* rearrangements in clinical practice to guide the appropriate therapy 9,10. So far, no reports have evaluated the association between *ALK* rearrangement status and imaging findings in advanced-stage lung adenocarcinoma. In this report, the imaging findings of 36 cases with advanced *ALK*-positive NSCLC were described. To the best of our knowledge, this is the first report describing the clinical features including the radiological findings and treatment responses to second-line treatment in advanced *ALK*-positive NSCLC patients. As to radiological findings, several studies have suggested that lung adenocarcinoma is significantly associated with GGO 11,12, and Aoki et al. reported that patients with GGO components of more than 50% showed a significantly better prognosis than those with GGO components of less than 50%. The tumors in a majority of the present cases showed a mass or a nodule with a solid pattern of growth in the radiological findings without GGO. Similar to the *ALK*-positive lung adenocarcinomas in an early stage 4, these features might suggest that they have a more invasive nature than those with more GGO components. In this study, a nodule (≤30 mm) was observed in 44.4%, indicating relatively small-sized tumors, and more than 80% of the patients had lymphadenopathy, 19.4% showed extranodal invasion of lymph nodes, and 8.3% had lymphangitis. In addition, a pleural effusion was seen in more than 40% of patients. These features suggest that *ALK*-positive lung cancer could have a tendency for the tumor to infiltrate into surrounding bronchovascular sheaths or localized lymphangitic extension. Further studies on the correlation between radiological appearance and the clinical features of advanced *ALK*-rearranged lung adenocarcinoma are warranted.

It has been reported that *ALK*-positive patients have lower rates of response to platinum-based chemotherapy than patients with *EGFR* mutations in the Caucasian population 1. In this study, a half of patients had PR, and 30% had SD, with relatively good response rates to cytotoxic drugs in the first-line chemotherapy. However, in the second-line chemotherapy, the patients treated with cytotoxic agents had no clinical response, although the patients who were treated by crizotinib showed good responses. Despite the limited number of cases, the present data suggest that *ALK*-positive patients have a good response rate to cytotoxic drugs in the first-line setting, but a relatively low response rate in the second-line setting. These data support the potential clinical benefit of using ALK inhibitors, at least as the second-line agents.

This study is limited by its retrospective nature. In addition, although 36 cases of advanced *ALK*-positive NSCLC identified in a single institution during a 6-year period were included, the number of cases included is still small. Moreover, evaluation of the response to chemotherapy was possible in only 20 patients. Further studies with a large number of cases are needed.

In conclusion, advanced-stage *ALK*-positive tumors were relatively small in size, but the majority of them exhibited lymphadenopathy and a solid growth pattern without GGO on CT. In addition, more than 40% of patients had a pleural effusion. Identification of the relationship between CT imaging findings and *ALK* molecular status can help define categories of lung adenocarcinoma that have distinct clinical, radiological, molecular, and pathological characteristics. These findings may help to make a proper diagnosis of *ALK*-positive NSCLC based on the radiological findings.
